# Supplementary material for: Predictive Factors for 24-h Survival After Perioperative Cardiopulmonary Resuscitation: Single-Center Retrospective Cohort Study
Source: J Clin Med. 2025 Jan 17;14(2):599. doi: 10.3390/jcm14020599 (PMC11766343; doi:10.3390/jcm14020599)
Supplement: Supplementary file 1 [file jcm-14-00599-s001.zip › Supplementary file S1.pdf]

Supplementary File S1. Details of the collected data including information about the hospital staff and their competencies.

The study was conducted at Chiang Mai University Hospital, a tertiary teaching hospital in Northern Thailand with 1,400 beds. Scope of hospital services over 16,000 surgeries for patients undergoing outpatient or inpatient diagnostic or surgical procedures, whether elective or emergency, during working hours (08:00 am–04:00 pm) or non-working hours (04:00 pm–08:00 am). Our department's service scope includes general, orthopedic, neurosurgery, vascular, urology, gynecology, pediatric, ear-nose-throat, and plastic surgery under general anesthesia (GA) with/without regional block or peripheral nerve block, regional anesthesia alone, and all sedated monitored anesthesia care (MAC). There is a 16-bed Post-Anesthesia Care Unit (PACU) nearby to the main OR, while the obstetrics units have their own PACUs. Anesthesia services are also provided for embolization therapy and radiology procedures. All patients were visited by their assigned anesthesiologist one day before surgery. This was followed by a 5-minute conference with the anesthesia team and operating room nurses on the morning of the surgery to plan the anesthesia. Additionally, all emergency patients underwent a rapid physical assessment by the on-duty anesthesiologist to ensure readiness before surgery.

The standards of the American Society of Anesthesiologists (ASA) for monitoring were used: electrocardiography (EKG), pulse oximetry (SpO_2), blood pressure, temperature, and end-tidal carbon dioxide ($ETCO_2$) measurements. Critically ill patients or those undergoing major surgery are subjected to invasive blood-pressure measurements and central venous access.

The Department of Anesthesiology at Chiang Mai University Hospital comprises 25 anesthesiologists, 54 anesthesia residents, 70 nurse anesthetists, and 36 nursing assistants. Records of anesthesia are maintained for all patients, including both inpatients and outpatients. Perioperative cardiac arrest (POCA) incidents are reported and documented. A Risk Management Committee, consisting of staff anesthesiologists, anesthesia chief residents, certified nurse anesthetists, and recovery room nurses (when incidents involve the PACU or patient transfer), meets monthly to review all incidents. Data from all POCA events are discussed in departmental meetings, where they are reviewed and root cause analyzed by two or three senior staff anesthesiologists. The POCA registry includes information such as patient demographics, the surgical and perioperative detail, intraoperative parameter, the detail of POCA events, cause of POCA and outcomes.

The Department of Anesthesiology maintains a database of all patients who experience perioperative cardiac arrest as part of the department's Risk Management Committee effort. Reporting all critical incidents by anesthesia providers is mandatory for this initiative.

All anesthesia providers at Chiang Mai University Hospital, the only medical school in northern Thailand, receive advanced CPR training according to American Heart Association (AHA) 2020 guidelines. The training comprises regular practice sessions for both basic and advanced life support every two years, supervised by certified ACLS instructors from the Thai Resuscitation Council, with an annual audit of CPR practices. Additionally, the hospital is developing a Rapid Response Team (RRT) and establishing protocols for quickly activating the team during emergencies requiring CPR. Key factors influencing patient survival rates

include prompt defibrillation and the presence of skilled, rapid responders, ensuring the highest standards of patient safety within the medical facility.
