# Supplementary material for: Predictive Factors for 24-h Survival After Perioperative Cardiopulmonary Resuscitation: Single-Center Retrospective Cohort Study
Source: J Clin Med. 2025 Jan 17;14(2):599. doi: 10.3390/jcm14020599 (PMC11766343; doi:10.3390/jcm14020599)
Supplement: Supplementary file 1 [file jcm-14-00599-s001.zip › Supplementary table S1.pdf]

Supplementary Table S1. Data measurement and Quantitative variable

Variable	Group	Reference
Age	<65 year<br>≥65 year	[1-3]
ASA	1-2<br>3-5	[1, 4-8]
Intraoperative blood loss	≤3,000 ml<br>>3,000 ml	[9]
Time of cardiac arrest	Working hours (7.31 am-3.30 pm)<br>Non-working hours (3.31 pm-7.30 am)	[10]
Initial rhythm at time of CPR	Shockable rhythm (VT/VF)<br>Non shockable rhythm (PEA/Asystole)	[9, 11-13]
Duration of CPR	≤ 30 minutes<br>> 30 minutes	[9]
Blood glucose level	<70 mg/dL<br>71-239 mg/dL<br>> 240 mg/dL	[14]
Hemoglobin	< 8 g/dL<br>≥ 8 g/dL	[15]
Platelet count	≤ 100,000 cell/µl<br>> 100,000 cell/µl	[16]
Neutrophil Lymphocyte ratio (NLR)	≤ 9<br>> 9	[17]
Serum albumin	≤ 3.5 g/dL<br>> 3.5 g/dL	[18]
Serum creatinine	≤ 2 mg/dL<br>> 2 mg/dL	[19]
Serum Sodium	<135 mEq/L<br>135-145 mEq/L<br>>145 mEq/L	[19, 20]
Potassium	< 3.5 mEq/L<br>3.5 – 4.9 mEq/L<br>> 5 mEq/L	[20, 21]
Heart rate (bpm)	>100 beats per minutes<br>51 – 100 beats per minutes<br>≤ 50 beats per minutes	[19]
Systolic Blood Pressure	>140 mmHg<br>81 – 140 mmHg<br>≤ 80 mmHg	[19]
Diastolic Blood Pressure	<50 mmHg<br>51 – 80 mmHg<br>> 80 mmHg	[22]
Mean Arterial Pressure (mmHg)	>95 mmHg<br>65 – 95 mmHg<br><65 mmHg	[22]
Oxygen saturation	≥ 90%<br>< 90%	[8, 10]
End-Tidal Carbon-dioxide	35 – 45 mmHg<br>< 35 mmHg	[23]

Reference

1. Braz L, Morais A, Sanchez R, Porto D, Pacchioni M, Serafim W, et al. Epidemiology of perioperative cardiac arrest and mortality in Brazil: systematic review. Brazilian Journal of Anesthesiology (English Edition). 2020;70.
2. Hanif AA, Rachman IA, Yuwono HS. Factors Influencing the Success Rate of Cardiopulmonary Resuscitation. Althea Medical Journal. 2015;2(4).
3. An J-x, Zhang L-M, Sullivan EA, Guo Q-l, Williams JP. Intraoperative cardiac arrest during anesthesia: a retrospective study of 218 274 anesthetics undergoing non-cardiac surgery in a US teaching hospital. Chinese Medical Journal. 2011;124(2).
4. Aloweidi A, Alghanem S, Bsisu I, Ababneh O, Alrabayah M, Al-Zaben K, et al. Perioperative Cardiac Arrest: A 3-Year Prospective Study from a Tertiary Care University Hospital. Drug, healthcare and patient safety. 2022;Volume 14:1-8.
5. Braz LG, Módolo NSP, do Nascimento P, Jr, Bruschi BAM, Castiglia YMM, Ganem EM, et al. Perioperative cardiac arrest: a study of 53 718 anaesthetics over 9 yr from a Brazilian teaching hospital. BJA: British Journal of Anaesthesia. 2006;96(5):569-75.
6. Newland MC, Ellis SJ, Lydiatt CA, Peters KR, Tinker JH, Romberger DJ, et al. Anesthetic-related cardiac arrest and its mortality: a report covering 72,959 anesthetics over 10 years from a US teaching hospital. Anesthesiology. 2002;97(1):108-15.
7. Sprung J, Warner ME, Contreras MG, Schroeder DR, Beighley CM, Wilson GA, et al. Predictors of survival following cardiac arrest in patients undergoing noncardiac surgery: a study of 518,294 patients at a tertiary referral center. Anesthesiology. 2003;99(2):259-69.
8. Braz LG, Braz JRC, Modolo MP, Corrente JE, Sanchez R, Pacchioni M, et al. Perioperative and anesthesia-related cardiac arrest and mortality rates in Brazil: A systematic review and proportion meta-analysis. PLoS One. 2020;15(11):e0241751.
9. Siriphuwanun V, Punjasawadwong Y, Lapisatepun W, Charuluxananan S, Uerpairojkit K, Patumanond J. The initial success rate of cardiopulmonary resuscitation and its associated factors in patients with cardiac arrest within 24 hours after anesthesia for an emergency surgery. Risk Manag Healthc Policy. 2014;7:65-76.
10. Siriphuwanun V, Punjasawadwong Y, Lapisatepun W, Charuluxananan S, Uerpairojkit K. Prognostic factors for death and survival with or without complications in cardiac arrest patients receiving CPR within 24 hours of anesthesia for emergency surgery. Risk Manag Healthc Policy. 2014;7:199-210.
11. Alao DO, Mohammed NA, Hukan YO, Al Neyadi M, Jummani Z, Dababneh EH, et al. The epidemiology and outcomes of adult in-hospital cardiac arrest in a high-income developing country. Resuscitation plus. 2022;10:100220.
12. Chomchoey C, Thawitsri T. The incidence of postoperative cardiac arrest and pre-resuscitation factors associated with post- cardiopulmonary resuscitation mortality: a single-center study in Thailand. Clinical Critical Care. 2021;29:2021:e0007.
13. Rukewe A, Fatiregun A, Osunlaja TO. Cardiac arrest during anesthesia at a university Hospital in Nigeria. Nigerian journal of clinical practice. 2014;17(1):28-31.
14. Wang C-H, Huang C-H, Chang W-T, Tsai M-S, Yu P-H, Wang A-Y, et al. Association between hemoglobin levels and clinical outcomes in adult patients after in-hospital cardiac arrest: a retrospective cohort study. Internal and Emergency Medicine. 2016;11(5):727-36.
15. Kim IJ, Yang PS, Kim TH, Uhm JS, Pak HN, Lee MH, et al. Relationship Between Anemia and the Risk of Sudden Cardiac Arrest - A Nationwide Cohort Study in South Korea. Circulation journal : official journal of the Japanese Circulation Society. 2018;82(12):2962-9.
16. Brown LM, Call MS, Margaret Knudson M, Cohen MJ, Holcomb JB, Wade CE, et al. A normal platelet count may not be enough: the impact of admission platelet count on

mortality and transfusion in severely injured trauma patients. The Journal of trauma. 2011;71(2 Suppl 3):S337-42.
17. Chae YJ, Lee J, Park JH, Han DG, Ha E, Yi IK. Late Mortality Prediction of Neutrophil-to-Lymphocyte and Platelet Ratio in Patients With Trauma Who Underwent Emergency Surgery: A Retrospective Study. The Journal of surgical research. 2021;267:755-61.
18. Hong SI, Kim YJ, Cho YJ, Huh JW, Hong SB, Kim WY. Predictive value of pre-arrest albumin level with GO-FAR score in patients with in-hospital cardiac arrest. Sci Rep. 2021;11(1):10631.
19. Barnett S, Moonesinghe SR. Clinical risk scores to guide perioperative management. Postgraduate medical journal. 2011;87(1030):535.
20. Vane MF, Carmona MJC, Pereira SM, Kern KB, Timerman S, Perez G, et al. Predictors and their prognostic value for no ROSC and mortality after a non-cardiac surgery intraoperative cardiac arrest: a retrospective cohort study. Scientific Reports. 2019;9(1):14975.
21. Yongyukantorn K, Oofuvong MJJoG, Geriatrics. Risk factors of intraoperative and 24-hour postoperative cardiac arrest in geriatric patients in non-cardiac surgery. 2020.
22. Han F, Wang Y, Wang Y, Dong J, Nie C, Chen M, et al. Intraoperative cardiac arrest: A 10-year study of patients undergoing tumorous surgery in a tertiary referral cancer center in China. Medicine (Baltimore). 2017;96(17):e6794-e.
23. Moitra V, Einav S, Thies K-C, Nunnally M, Gabrielli A, Maccioli G, et al. Cardiac Arrest in the Operating Room: Resuscitation and Management for the Anesthesiologist Part 1. Anesthesia & Analgesia. 2018;126:87V 888.
