# Supplementary material for: Predictive Factors for 24-h Survival After Perioperative Cardiopulmonary Resuscitation: Single-Center Retrospective Cohort Study
Source: J Clin Med. 2025 Jan 17;14(2):599. doi: 10.3390/jcm14020599 (PMC11766343; doi:10.3390/jcm14020599)
Supplement: Supplementary file 1 [file jcm-14-00599-s001.zip › Supplementary table S2.pdf]

Supplementary Table S2. Subgroup analysis between Age and trauma surgery (n=288)

Age	Trauma	Non-Trauma
< 65 years	128(67.02%)	63(32.98%)
≥ 65 years	21(21.65%)	76(78.35%)
