# Supplementary material for: Predictive Factors for 24-h Survival After Perioperative Cardiopulmonary Resuscitation: Single-Center Retrospective Cohort Study
Source: J Clin Med. 2025 Jan 17;14(2):599. doi: 10.3390/jcm14020599 (PMC11766343; doi:10.3390/jcm14020599)
Supplement: Supplementary file 1 [file jcm-14-00599-s001.zip › Supplementary table S3.pdf]

Supplementary Table S3. Subgroup analysis between age and Type of surgery (n=288)

**Type of surgery**	**Age ≥ 65** (97)	**Age < 65** (191)
Intrathoracic surgery	5(5.15%)	32(16.75%)
Intraabdominal surgery (Major vascular)	43(44.33%)	12(6.28%)
Intraabdominal surgery (General)	31(31.96%)	69(36.13%)
Orthopedic surgery	1(1.03%)	3(1.57%)
Intracranial surgery	7(7.22%)	45(23.56%)
The other surgery	9(9.28%)	17(8.90%)
Multiple injuries	1(1.03%)	13(6.81%)

Note: Other surgeries include procedures involving the eyes, ears, and throat. Multiple injuries refer to cases requiring more than one type of surgery
