# Supplementary material for: Predictive Factors for 24-h Survival After Perioperative Cardiopulmonary Resuscitation: Single-Center Retrospective Cohort Study
Source: J Clin Med. 2025 Jan 17;14(2):599. doi: 10.3390/jcm14020599 (PMC11766343; doi:10.3390/jcm14020599)
Supplement: Supplementary file 1 [file jcm-14-00599-s001.zip › Supplementary table S4.pdf]

Supplementary Table S4. Subgroup analysis between age and type of operations in Trauma group (149 patients)

Type of surgery	< 65 years (128)		≥ 65 years (21)	
	**Survival** 13(10.16%)	**Death** 115(89.84%)	**Survival** 8(38.10%)	**Death** 13(61.90%)
Intrathoracic surgery	2(15.38%)	19(16.52%)	0	2(15.38%)
Intraabdominal surgery (Major vascular)	0	2(1.74%)	0	1(7.69%)
Intraabdominal surgery (General)	4(30.77%)	39(33.91%)	3(37.50%)	5(38.46%)
Orthopedic surgery	0	2(1.74%)	0	1(7.69%)
Intracranial surgery	4(30.77%)	33(28.70%)	3(37.50%)	3(23.08%)
The other surgery	2(15.38%)	8(6.96%)	2(25.00%)	0
Multiple injuries	1(7.69%)	12(10.43%)	0	1(7.69%)

Note: Other surgeries include procedures involving the eyes, ears, and throat. Multiple injuries refer to cases requiring more than one type of surgery
